# 802. Investigation of a Novel Intraoperative Fluorescence Imaging Technique for Burn Excision

**DOI:** 10.1093/jbcr/irag033.180

**Published:** 2026-04-06

**Authors:** Mary Junak, Hector Garcia, Aiping Liu, Bailey Donahue, Adam J Uselmann, Lee D Faucher, Brian Pogue, Angela Gibson

**Affiliations:** University of Wisconsin Burn and Wound Center, Madison, WI; Dartmouth, Hanover, NH; University of Wisconsin Burn and Wound Center, Madison, WI; University of Wisconsin Burn and Wound Center, Madison, WI; OnLume Inc., Madison, WI; OnLume Inc., Madison, WI; University of Wisconsin Burn and Wound Center, Madison, WI

## Abstract

**Introduction:**

Interpatient heterogeneity was identified in an ongoing human subjects study investigating the use of indocyanine green (ICG) fluorescence imaging to evaluate burn depth and healing potential. A parallel swine model was initiated to investigate Second Window Indocyanine Green (SWIG), a novel method of delayed fluorescence imaging, in burn depth diagnosis and intraoperative visualization of excision endpoint.

**Methods:**

Adult pigs with burns of various depths received a 7 mg injection of ICG for angiography (ICGA) followed by a 5 mg/kg intravenous infusion of ICG on post-burn day (PBD) 1 or 2. SWIG imaging was performed the following day on a region of interest (ROI). ICG microscopy and histologic staining was performed for tissue architecture and viability on a full thickness biopsy of the ROI. Fluorescence signal was captured before and after tangential excision. GraphPad Prism 8.0 was used for statistical analyses with Spearman correlation coefficient (ρ) due to the non-normal distribution of the data.

**Results:**

There was a strong inverse correlation between perfusion as measured by ICGA peak fluorescence intensity and histologic burn depth (ρ = -0.89). SWIG signal-to-background ratio (SBR) was inversely correlated to histologic burn depth such that deeper burns exhibited less SWIG fluorescence signal than more superficial burns (ρ = -0.56). SWIG SBR was positively correlated with ICGA parameters including ICGA peak fluorescence intensity (ρ = 0.73) and ICGA egress slope (ρ = 0.67). In partial-thickness burns, SWIG fluorescence intensity progressively decreased with sequential excisions and was absent once a healthy, well-perfused wound bed was exposed. Deeper burns initially demonstrated low SWIG; however, the fluorescence increased with each excision until signal was again absent at the level of viable tissue (Fig. 1).

**Conclusions:**

Variability in perfusion and inflammation contributes to heterogeneity in the ICGA and SWIG parameters in patients. The swine model data suggests that permeability and vascular destruction effects ICG delivery to the wound and ultimately, the SWIG signal. Furthermore, the eschar in deeper burns in pig skin interferes with top-down visualization of the deeper fluorescence signal and contributes to variable results. However, visualization of SWIG fluorescence during tangential excision to determine endpoints of excision may represent a novel use of this technique. Parallel studies of human and swine models more rapidly uncover the challenges with introducing new technologies and may facilitate translation through iterative experimental design approaches.

**Applicability of Research to Practice:**

Our revised experimental approach has revealed that vascular destruction and eschar formation impair ICG delivery and fluorescence signal detection, highlighting key opportunities to address prior to translation for clinical use.

**Funding for the study:**

NIGMS R01GM145723.

